# Supplementary material for: Anesthesia for non-obstetric surgery during late term pregnancy in mares
Source: PLoS One. 2024 Nov 22;19(11):e0313563. doi: 10.1371/journal.pone.0313563 (PMC11584139; doi:10.1371/journal.pone.0313563)
Supplement: S1 Table — Mean and standard deviation of temperature (°C), respiratory rate (rpm), heart rate (bpm), end-tidal carbon dioxide (EtCO2), oxygen saturation (SpO2), systolic (SAP; mmHg), mean (MAP; mmHg) and diastolic (DAP; mmHg) arterial pressure during general inhalation anesthesia of mares in the last month of gestation. (DOCX) [file pone.0313563.s001.docx]

**S1 Table.** **General inhalation anesthesia parameters of mares in the last month of gestation.** Mean and standard deviation of temperature (^o^C), respiratory rate (rpm), heart rate (bpm), end-tidal carbon dioxide (EtCO_2_), oxygen saturation (SpO_2_), systolic (SAP; mmHg), mean (MAP; mmHg) and diastolic (DAP; mmHg) arterial pressure during general inhalation anesthesia of mares in the last month of gestation.

| **Time** | **Temperature**  **(0C)** | **Respiratory rate (rpm)** | **Heart rate**  **(bpm)** | **ETCO2** | **SpO2** | **SAP**  **(mmHg)** | **MAP**  **(mmHg)** | **DAP**  **(mmHg)** |
| --- | --- | --- | --- | --- | --- | --- | --- | --- |
| **15** | 37.40±0.36 a | 9.78±2.39 b | 48.33±7.97 b | 38.89±9.05 a | 95.11±2.85 b | 68.43±3.64 b | 43.43±2.15 b | 27.29±3.82 b |
| **25** | 37.29±0.45 a | 10.78±2.73 ab | 69.56±20.83 a | 41.00±7.12 a | 96.44±3.05 ab | 76.13±14.54 ab | 52.63±9.27 a | 38.00±6.63 a |
| **35** | 37.22±0.48 ab | 9.56±1.51 ab | 75.22±13.80 a | 49.33±9.26 a | 96.67±2.50 ab | 76.38±8.35 ab | 56.38±3.66 a | 38.88±3.83 a |
| **45** | 37.08±0.48 abc | 10.11±1.27 ab | 80.67±13.85 a | 47.00±7.00 a | 97.56±2.88 a | 74.00±6.63 ab | 54.67±5.22 a | 39.44±2.83 a |
| **55** | 36.89±0.40 bcd | 10.33±1.73 ab | 80.00±15.52 a | 49.56±9.59 a | 97.67±2.29 a | 78.33±6.78 ab | 58.56±5.94 a | 42.11±7.10 a |
| **65** | 36.83±0.33cd | 11.22±2.82 ab | 76.22±16.54 a | 48.33±7.16 a | 97.89±1.96 a | 77.89±9.51 ab | 56.78±3.38 a | 45.33±10.49 a |
| **75** | 36.73±0.38 cd | 11.67±3.57 ab | 76.78±15.34 a | 41.89±11.41 a | 97.89±2.67 a | 80.33±12.20 a | 60.22±6.20 a | 46.11±6.13 a |
| **85** | 36.67±0.52cd | 10.83±2.64 ab | 65.17±14.80 a | 43.33±11.33 a | 96.50±3.27 ab | 80.00±9.72 ab | 57.00±5.15 a | 41.20±6.98 a |
| **90** | 36.58±0.51 d | 12.17±2.48 a | 66.50±21.70 a | 46.00±7.87 a | 98.17±0.75 a | 79.67±11.52 ab | 57.50±4.76 a | 43.33±3.56 a |

*a-b-c-d uncommon superscripts letters differ significantly (p< 0.05).
