# Supplementary material for: Anesthesia for non-obstetric surgery during late term pregnancy in mares
Source: PLoS One. 2024 Nov 22;19(11):e0313563. doi: 10.1371/journal.pone.0313563 (PMC11584139; doi:10.1371/journal.pone.0313563)
Supplement: S2 Table — Mean and standard deviation of pH, pCO2 (mmHg), pO2 (mmHg), base excess (mmol/L), HCO3 (mmol/L), tCO2 (mmol/L), SO2 (%), sodium (mmol/L), potassium (mmol/L), calcium (mmol/L), and lactate (mmol/L) during general inhalation anesthesia of mares in the last month of gestation. (DOCX) [file pone.0313563.s002.docx]

**S2 Table.** **General inhalation anesthesia hemogasometry of mares in the last month of gestation.** Mean and standard deviation of pH, pCO_2_ (mmHg), pO_2_ (mmHg), base excess (mmol/L), HCO_3_ (mmol/L), tCO_2_ (mmol/L), SO_2_ (%), sodium (mmol/L), potassium (mmol/L), calcium (mmol/L), and lactate (mmol/L) during general inhalation anesthesia of mares in the last month of gestation.

| **Time** | **pH** | **pCO_2_**  **(mmHg)** | **pO_2_**  **(mmHg)** | **Base excess**  **(mmol/L)** | **HCO_3_**  **(mmol/L)** | **tCO_2_**  **(mmol/L)** | **SO2**  **(%)** | **Sodium**  **(mmol/L)** | **Potassium**  **(mmol/L)** | **Calcium**  **(mmol/L)** | **Lactate**  **(mmol/L)** |
| --- | --- | --- | --- | --- | --- | --- | --- | --- | --- | --- | --- |
| **0** | 7.33±0.08 a | 50.51±8.89 b | 89.00±40.47 b | 0.50±3.59 a | 26.33±2.84 a | 27.88±2.90 a | 91.00±7.91 a | 138.75±1.49 a | 3.49±0.22 a | 0.95±0.13 a | 3.14±1.45 a |
| **45** | 7.27±0.05 ab | 58.80±6.26 ab | 139.88±80.27 a | 0.13±3.87 a | 27.01±3.19 a | 28.75±3.37 a | 95.50±6.48 a | 137.13±1.73 b | 3.21±0.22 b | 0.84±0.17 a | 3.33±1.25 a |
| **75** | 7.24±0.04 b | 62.55±5.07 a | 140.75±56.45 a | -0.13±3.80 a | 27.33±3.36 a | 29.25±3.45 a | 96.75±3.54 a | 136.38±2.13 bc | 3.29±0.24 b | 0.87±0.13 a | 3.38±1.01 a |
| **90** | 7.24±0.06 b | 60.34±6.25 ab | 128.88±53.22 ab | -1.75±4.56 a | 25.89±3.80 a | 27.75±4.03 a | 95.75±4.50 a | 135.88±2.10 c | 3.24±0.22 b | 0.82±0.16 a | 3.43±0.79 a |

a-b-c-d uncommon superscripts letters differ significantly (p< 0.05).
