# Supplementary material for: Anesthesia for non-obstetric surgery during late term pregnancy in mares
Source: PLoS One. 2024 Nov 22;19(11):e0313563. doi: 10.1371/journal.pone.0313563 (PMC11584139; doi:10.1371/journal.pone.0313563)
Supplement: S3 Table — Mean and standard deviation of pulmonary arterial pressure (PAP; mmHg), central venous pressure (CVP; mmHg), cardiac output (CO; L), and cardiac index (CI; mL) during general inhalation anesthesia of mares in the last month of gestation. (DOCX) [file pone.0313563.s003.docx]

**S3 Table.** **General inhalation anesthesia hemodynamic of mares in the last month of gestation**. Mean and standard deviation of pulmonary arterial pressure (PAP; mmHg), central venous pressure (CVP; mmHg), cardiac output (CO; L), and cardiac index (CI; mL) during general inhalation anesthesia of mares in the last month of gestation.

| **Time** | **PAP (mmHg)** | **CPV (mmHg)** | **CO (L)** | **CI (mL)** |
| --- | --- | --- | --- | --- |
| **0** | 23.71±8.01 a | 8.86±8.59 a | 37.12±5.71 a | 93.17±13.59 a |
| **15** | 10.88±4.09 b | 1.63±8.72 ab | 28.63±11.73 a | 73.65±35.63 a |
| **25** | 13.25±4.03 b | 1.86±10.35 ab | 31.73±7.18 a | 78.35±17.65 a |
| **35** | 8.13±8.51 b | 1.88±11.72 ab | 38.00±10.00 a | 95.80±26.06 a |
| **45** | 11.43±4.73 b | -1.38±4.57 b | 38.11±9.77 a | 95.67±23.57 a |
| **60** | 8.63±5.10 b | -1.00±4.31 b | 33.56±9.22 a | 84.66±24.18 a |
| **75** | 7.50±2.73 b | -1.50±4.24 b | 31.78±10.18 a | 80.26±26.49 a |
| **90** | 7.75±5.31 b | 1.63±6.99 ab | 29.04±7.52 a | 73.57±22.49 a |

*a-b-c-d uncommon superscripts letters differ significantly (p< 0.05).
