# Supplementary material for: Anesthesia for non-obstetric surgery during late term pregnancy in mares
Source: PLoS One. 2024 Nov 22;19(11):e0313563. doi: 10.1371/journal.pone.0313563 (PMC11584139; doi:10.1371/journal.pone.0313563)
Supplement: S4 Table — Mean and standard deviation of stroke volume (SV; mL/beats) and peripheric vascular resistance (PVR) during general inhalation anesthesia of mares in the last month of gestation. (DOCX) [file pone.0313563.s004.docx]

**S4 Table.** **General inhalation anesthesia hemodynamic of mares in the last month of gestation**. Mean and standard deviation of stroke volume (SV; mL/beats) and peripheric vascular resistance (PVR) during general inhalation anesthesia of mares in the last month of gestation.

| **Time** | **SV (mL/beats)** | **PVR** |
| --- | --- | --- |
| **15** | 598.10±175.26 a | 132.02±51.60 a |
| **25** | 343.40±185.27 b | 136.17±56.32 a |
| **35** | 441.88±124.91 ab | 126.12±52.92 a |
| **45** | 432.08±117.89 ab | 127.55±36.40 a |
| **75** | 398.87±110.89 b | 165.35±53.60 a |
| **90** | 319.73±173.67 b | 182.08±35.21 a |

*a-b-c-d uncommon superscripts letters differ significantly (p< 0.05).
