# Supplementary material for: Anesthesia for non-obstetric surgery during late term pregnancy in mares
Source: PLoS One. 2024 Nov 22;19(11):e0313563. doi: 10.1371/journal.pone.0313563 (PMC11584139; doi:10.1371/journal.pone.0313563)
Supplement: S5 Table — Mean and standard deviation of fetal heart rate (bpm) during general inhalation anesthesia of mares in the last month of gestation. (DOCX) [file pone.0313563.s005.docx]

**S5 Table.** **Fetal heart rate.** Mean and standard deviation of fetal heart rate (bpm) during general inhalation anesthesia of mares in the last month of gestation.

| **Time** | **Fetal US (bpm)** |
| --- | --- |
| **0** | 84.50±12.07 a |
| **15** | 73.63±8.31 a |
| **25** | 65.13±7.08 b |
| **35** | 62.38±7.05 b |
| **45** | 62.88±7.49 b |
| **60** | 60.38±5.66 b |
| **75** | 60.75±5.92 b |
| **Tpost** | 78.11±14.82 a |

*a-b-c-d uncommon superscripts letters differ significantly (p< 0.05).
