# Supplementary material for: Anesthesia for non-obstetric surgery during late term pregnancy in mares
Source: PLoS One. 2024 Nov 22;19(11):e0313563. doi: 10.1371/journal.pone.0313563 (PMC11584139; doi:10.1371/journal.pone.0313563)
Supplement: S6 Table — Maternal heart rate. Maternal heart rate (bpm) during general inhalation anesthesia and dorsal recumbency of mares in the last month of gestation. (DOCX) [file pone.0313563.s006.docx]

| **Mare Heart Rate (bpm)** | | | | | | | | | | | |
| --- | --- | --- | --- | --- | --- | --- | --- | --- | --- | --- | --- |
| **Time (minutes)** | **Horse 1** | **Horse 2** | **Horse 3** | **Horse 4** | **Horse 5** | **Horse 6** | **Horse 7** | **Horse 8** | **Horse 9** | **Mean** | **SD** |
| **T15** | 54 | 60 | 37 | 46 | 57 | 51 | 41 | 49 | 40 | 48,33 | 7,97 |
| **T25** | 70 | 67 | 70 | 83 | 81 | 110 | 40 | 58 | 47 | 69,56 | 20,83 |
| **T35** | 79 | 77 | 83 | 90 | 87 | 86 | 60 | 65 | 50 | 75,22 | 13,80 |
| **T45** | 73 | 76 | 87 | 94 | 91 | 89 | 79 | 88 | 49 | 80,67 | 13,85 |
| **T55** | 82 | 72 | 70 | 98 | 90 | 88 | 84 | 90 | 46 | 80,00 | 15,52 |
| **T65** | 83 | 71 | 64 | 86 | 77 | 85 | 91 | 90 | 39 | 76,22 | 16,54 |
| **T75** | 81 | 84 | 62 | 82 | 82 | 89 | 79 | 90 | 42 | 76,78 | 15,34 |
| **T85** | 69 | 64 | 53 | 83 | - | - | 78 | - | 44 | 65,17 | 14,80 |
| **T90** | - | - | 50 | 71 | 71 | 103 | - | 64 | 40 | 66,50 | 21,70 |

**S6 Table. Raw Data. Maternal heart rate.** Maternal heart rate (bpm) during general inhalation anesthesia and dorsal recumbency of mares in the last month of gestation.
