# Supplementary material for: Anesthesia for non-obstetric surgery during late term pregnancy in mares
Source: PLoS One. 2024 Nov 22;19(11):e0313563. doi: 10.1371/journal.pone.0313563 (PMC11584139; doi:10.1371/journal.pone.0313563)
Supplement: S7 Table — Maternal respiratory rate. Maternal respiratory rate (rpm) during general inhalation anesthesia and dorsal recumbency of mares in the last month of gestation. (DOCX) [file pone.0313563.s007.docx]

**S7 Table. Raw Data. Maternal respiratory rate.** Maternal respiratory rate (rpm) during general inhalation anesthesia and dorsal recumbency of mares in the last month of gestation.

| **Respiratory Rate (rpm)** | | | | | | | | | | | |
| --- | --- | --- | --- | --- | --- | --- | --- | --- | --- | --- | --- |
| **Time (minutes)** | **Horse 1** | **Horse 2** | **Horse 3** | **Horse 4** | **Horse 5** | **Horse 6** | **Horse 7** | **Horse 8** | **Horse 9** | **Mean** | **SD** |
| **T15** | 13 | 7 | 9 | 9 | 9 | 10 | 7 | 14 | 10 | 9,78 | 2,39 |
| **T25** | 15 | 7 | 9 | 10 | 9 | 15 | 10 | 10 | 12 | 10,78 | 2,73 |
| **T35** | 11 | 9 | 9 | 9 | 9 | 11 | 9 | 7 | 12 | 9,56 | 1,51 |
| **T45** | 10 | 10 | 9 | 9 | 13 | 10 | 9 | 10 | 11 | 10,11 | 1,27 |
| **T55** | 10 | 9 | 14 | 10 | 12 | 10 | 10 | 8 | 10 | 10,33 | 1,73 |
| **T65** | 14 | 7 | 14 | 9 | 15 | 13 | 10 | 9 | 10 | 11,22 | 2,82 |
| **T75** | 14 | 9 | 20 | 10 | 9 | 13 | 10 | 10 | 10 | 11,67 | 3,57 |
| **T85** | 9 | 8 | 15 | 9 | - | - | 12 | - | 12 | 10,83 | 2,64 |
| **T90** | - | - | 13 | 9 | 10 | 13 | - | 16 | 12 | 12,17 | 2,48 |
