# Supplementary material for: Anesthesia for non-obstetric surgery during late term pregnancy in mares
Source: PLoS One. 2024 Nov 22;19(11):e0313563. doi: 10.1371/journal.pone.0313563 (PMC11584139; doi:10.1371/journal.pone.0313563)
Supplement: S8 Table — Maternal mean arterial pressure. Maternal mean arterial pressure (mmHg) during general inhalation anesthesia and dorsal recumbency of mares in the last month of gestation. (DOCX) [file pone.0313563.s008.docx]

**S8 Table. Raw Data. Maternal mean arterial pressure.** Maternal mean arterial pressure (mmHg) during general inhalation anesthesia and dorsal recumbency of mares in the last month of gestation.

| **Mean Arterial Pressure (mmHg)** | | | | | | | | | | | |
| --- | --- | --- | --- | --- | --- | --- | --- | --- | --- | --- | --- |
| **Time (minutes)** | **Horse 1** | **Horse 2** | **Horse 3** | **Horse 4** | **Horse 5** | **Horse 6** | **Horse 7** | **Horse 8** | **Horse 9** | **Mean** | **SD** |
| **T15** | - | 42 | 46 | 43 | 40 | 44 | - | 43 | 46 | 43,43 | 2,15 |
| **T25** | - | 50 | 60 | 60 | 64 | 50 | 34 | 51 | 52 | 52,63 | 9,27 |
| **T35** | - | 57 | 60 | 59 | 56 | 56 | 49 | 54 | 60 | 56,38 | 3,66 |
| **T45** | 45 | 50 | 55 | 53 | 62 | 55 | 57 | 61 | 54 | 54,67 | 5,22 |
| **T55** | 74 | 54 | 57 | 55 | 58 | 58 | 57 | 57 | 57 | 58,56 | 5,94 |
| **T65** | 52 | 55 | 63 | 57 | 60 | 55 | 59 | 56 | 54 | 56,78 | 3,38 |
| **T75** | 74 | 62 | 56 | 57 | 66 | 55 | 57 | 57 | 58 | 60,22 | 6,20 |
| **T85** | - | 52 | 62 | 59 | - | - | 51 | - | 61 | 57,00 | 5,15 |
| **T90** | - | - | 64 | 59 | 60 | 51 | - | 58 | 53 | 57,50 | 4,76 |
