# Supplementary material for: Anesthesia for non-obstetric surgery during late term pregnancy in mares
Source: PLoS One. 2024 Nov 22;19(11):e0313563. doi: 10.1371/journal.pone.0313563 (PMC11584139; doi:10.1371/journal.pone.0313563)
Supplement: S9 Table — Maternal diastolic arterial pressure. Maternal diastolic arterial pressure (mmHg) during general inhalation anesthesia and dorsal recumbency of mares in the last month of gestation. (DOCX) [file pone.0313563.s009.docx]

**S9 Table. Raw Data. Maternal diastolic arterial pressure.** Maternal diastolic arterial pressure (mmHg) during general inhalation anesthesia and dorsal recumbency of mares in the last month of gestation.

| **Diastolic Arterial Pressure (mmHg)** | | | | | | | | | | | |
| --- | --- | --- | --- | --- | --- | --- | --- | --- | --- | --- | --- |
| **Time (minutes)** | **Horse 1** | **Horse 2** | **Horse 3** | **Horse 4** | **Horse 5** | **Horse 6** | **Horse 7** | **Horse 8** | **Horse 9** | **Mean** | **SD** |
| **T15** | - | 26 | 31 | 28 | 23 | 22 | - | 29 | 32 | 27,29 | 3,82 |
| **T25** | - | 38 | 43 | 40 | 46 | 35 | 24 | 37 | 41 | 38,00 | 6,63 |
| **T35** | - | 40 | 42 | 43 | 36 | 39 | 35 | 33 | 43 | 38,88 | 3,83 |
| **T45** | 40 | 34 | 39 | 39 | 43 | 37 | 41 | 43 | 39 | 39,44 | 2,83 |
| **T55** | 58 | 31 | 43 | 40 | 38 | 42 | 44 | 42 | 41 | 42,11 | 7,10 |
| **T65** | 70 | 34 | 51 | 41 | 40 | 39 | 43 | 48 | 42 | 45,33 | 10,49 |
| **T75** | 60 | 41 | 47 | 40 | 44 | 44 | 43 | 51 | 45 | 46,11 | 6,13 |
| **T85** | - | 32 | 48 | 41 | - | - | 37 | - | 48 | 41,20 | 6,98 |
| **T90** | - | - | 50 | 43 | 42 | 44 | - | 40 | 41 | 43,33 | 3,56 |
