# Supplementary material for: Anesthesia for non-obstetric surgery during late term pregnancy in mares
Source: PLoS One. 2024 Nov 22;19(11):e0313563. doi: 10.1371/journal.pone.0313563 (PMC11584139; doi:10.1371/journal.pone.0313563)
Supplement: S10 Table — Maternal systolic arterial pressure. Maternal systolic arterial pressure (mmHg) during general inhalation anesthesia and dorsal recumbency of mares in the last month of gestation. (DOCX) [file pone.0313563.s010.docx]

**S10 Table. Raw Data. Maternal systolic arterial pressure.** Maternal systolic arterial pressure (mmHg) during general inhalation anesthesia and dorsal recumbency of mares in the last month of gestation.

| **Systolic Arterial Pressure (mmHg)** | | | | | | | | | | | |
| --- | --- | --- | --- | --- | --- | --- | --- | --- | --- | --- | --- |
| **Time (minutes)** | **Horse 1** | **Horse 2** | **Horse 3** | **Horse 4** | **Horse 5** | **Horse 6** | **Horse 7** | **Horse 8** | **Horse 9** | **Mean** | **SD** |
| **T15** | - | 72 | 74 | 66 | 68 | 68 | - | 63 | 68 | 68,43 | 3,64 |
| **T25** | - | 69 | 92 | 82 | 92 | 65 | 49 | 80 | 80 | 76,13 | 14,54 |
| **T35** | - | 74 | 84 | 71 | 80 | 78 | 64 | 70 | 90 | 76,38 | 8,35 |
| **T45** | 73 | 68 | 73 | 66 | 87 | 72 | 69 | 78 | 80 | 74,00 | 6,63 |
| **T55** | 90 | 79 | 80 | 69 | 81 | 82 | 71 | 71 | 82 | 78,33 | 6,78 |
| **T65** | 88 | 87 | 87 | 75 | 85 | 77 | 67 | 62 | 73 | 77,89 | 9,51 |
| **T75** | 91 | 93 | 84 | 80 | 96 | 68 | 65 | 64 | 82 | 80,33 | 12,20 |
| **T85** | - | 79 | 82 | 82 | - | - | 65 | - | 92 | 80,00 | 9,72 |
| **T90** | - | - | 90 | 85 | 92 | 67 | - | 65 | 79 | 79,67 | 11,52 |
