# Supplementary material for: Anesthesia for non-obstetric surgery during late term pregnancy in mares
Source: PLoS One. 2024 Nov 22;19(11):e0313563. doi: 10.1371/journal.pone.0313563 (PMC11584139; doi:10.1371/journal.pone.0313563)
Supplement: S11 Table — Maternal temperature. Maternal temperature (°C) during general inhalation anesthesia and dorsal recumbency of mares in the last month of gestation. (DOCX) [file pone.0313563.s011.docx]

**S11 Table. Raw Data. Maternal temperature.** Maternal temperature (^o^C) during general inhalation anesthesia and dorsal recumbency of mares in the last month of gestation.

| **Temperature (^o^C)** | | | | | | | | | | | |
| --- | --- | --- | --- | --- | --- | --- | --- | --- | --- | --- | --- |
| **Time (minutes)** | **Horse 1** | **Horse 2** | **Horse 3** | **Horse 4** | **Horse 5** | **Horse 6** | **Horse 7** | **Horse 8** | **Horse 9** | **Mean** | **SD** |
| **T15** | 37,2 | 37,6 | 36,7 | 37,5 | 38 | 37,5 | 37,6 | 37,3 | 37,2 | 37,40 | 0,36 |
| **T25** | 37 | 37,2 | 36,9 | 37,5 | 37,9 | 37,3 | 37,8 | 37,5 | 36,5 | 37,29 | 0,45 |
| **T35** | 37 | 37,1 | 37,4 | 37,5 | 37,9 | 37 | 37,4 | 37,5 | 36,2 | 37,22 | 0,48 |
| **T45** | 36,7 | 37,3 | 37,4 | 37,5 | 37,6 | 36,8 | 37 | 37,3 | 36,1 | 37,08 | 0,48 |
| **T55** | 36,7 | 37,2 | 37 | 37,5 | 37 | 36,6 | 36,8 | 37,1 | 36,1 | 36,89 | 0,40 |
| **T65** | 36,7 | 37,2 | 37 | 37,1 | 36,9 | 36,6 | 37 | 36,9 | 36,1 | 36,83 | 0,33 |
| **T75** | 36,7 | 37,1 | 36,7 | 37,1 | 37 | 36,5 | 37 | 36,6 | 35,9 | 36,73 | 0,38 |
| **T85** | 36,4 | 37,2 | 36,6 | 37,1 | - | - | 36,9 | - | 35,8 | 36,67 | 0,52 |
| **T90** | - | - | 36,6 | 37,2 | 36,8 | 36,4 | - | 36,8 | 35,7 | 36,58 | 0,51 |
