# Supplementary material for: Anesthesia for non-obstetric surgery during late term pregnancy in mares
Source: PLoS One. 2024 Nov 22;19(11):e0313563. doi: 10.1371/journal.pone.0313563 (PMC11584139; doi:10.1371/journal.pone.0313563)
Supplement: S12 Table — Maternal pO2. Maternal pO2 (mmHg) during general inhalation anesthesia and dorsal recumbency of mares in the last month of gestation. (DOCX) [file pone.0313563.s012.docx]

**S12 Table. Raw Data. Maternal pO_2_.** Maternal pO_2_ (mmHg) during general inhalation anesthesia and dorsal recumbency of mares in the last month of gestation.

| **paO_2_ (mmHg)** | | | | | | | | | | | |
| --- | --- | --- | --- | --- | --- | --- | --- | --- | --- | --- | --- |
| **Time (minutes)** | **Horse 1** | **Horse 2** | **Horse 3** | **Horse 4** | **Horse 5** | **Horse 6** | **Horse 7** | **Horse 8** | **Horse 9** | **Mean** | **SD** |
| **T15** | - | 54 | 158 | 59 | 106 | 139 | 71 | 57 | 68 | 89,00 | 40,47 |
| **T45** | - | 57 | 311 | 104 | 177 | 172 | 113 | 88 | 97 | 139,88 | 80,27 |
| **T75** | - | 127 | 199 | 171 | 193 | 200 | 77 | 81 | 78 | 140,75 | 56,45 |
| **T90** | - | 110 | 160 | 174 | 179 | 193 | 72 | 75 | 68 | 128,88 | 53,22 |
