# Supplementary material for: Anesthesia for non-obstetric surgery during late term pregnancy in mares
Source: PLoS One. 2024 Nov 22;19(11):e0313563. doi: 10.1371/journal.pone.0313563 (PMC11584139; doi:10.1371/journal.pone.0313563)
Supplement: S13 Table — Maternal pCO2. Maternal pCO2 (mmHg) during general inhalation anesthesia and dorsal recumbency of mares in the last month of gestation. (DOCX) [file pone.0313563.s013.docx]

**S13 Table. Raw Data. Maternal pCO_2_.** Maternal pCO_2_ (mmHg) during general inhalation anesthesia and dorsal recumbency of mares in the last month of gestation.

| **paCO_2_ (mmHg)** | | | | | | | | | | | |
| --- | --- | --- | --- | --- | --- | --- | --- | --- | --- | --- | --- |
| **Time (minutes)** | **Horse 1** | **Horse 2** | **Horse 3** | **Horse 4** | **Horse 5** | **Horse 6** | **Horse 7** | **Horse 8** | **Horse 9** | **Mean** | **SD** |
| **T15** | - | 62 | 43,2 | 49,9 | 36,4 | 54,8 | 44,2 | 52,9 | 60,7 | 50,51 | 8,89 |
| **T45** | - | 56,5 | 45,8 | 64 | 60,2 | 65,1 | 55,2 | 62,4 | 61,2 | 58,80 | 6,26 |
| **T75** | - | 52,9 | 57,7 | 61,4 | 65,7 | 63 | 65,3 | 66,6 | 67,8 | 62,55 | 5,07 |
| **T90** | - | 51 | 69,1 | 60,6 | 59 | 65,1 | 65,7 | 52,9 | 59,3 | 60,34 | 6,25 |
