# Supplementary material for: Anesthesia for non-obstetric surgery during late term pregnancy in mares
Source: PLoS One. 2024 Nov 22;19(11):e0313563. doi: 10.1371/journal.pone.0313563 (PMC11584139; doi:10.1371/journal.pone.0313563)
Supplement: S14 Table — Maternal pH. Maternal pH during general inhalation anesthesia and dorsal recumbency of mares in the last month of gestation. (DOCX) [file pone.0313563.s014.docx]

**S14 Table. Raw Data. Maternal pH.** Maternal pH during general inhalation anesthesia and dorsal recumbency of mares in the last month of gestation.

| **pH** | | | | | | | | | | | |
| --- | --- | --- | --- | --- | --- | --- | --- | --- | --- | --- | --- |
| **Time (minutes)** | **Horse 1** | **Horse 2** | **Horse 3** | **Horse 4** | **Horse 5** | **Horse 6** | **Horse 7** | **Horse 8** | **Horse 9** | **Mean** | **SD** |
| **T15** | - | 7,17 | 7,36 | 7,37 | 7,45 | 7,34 | 7,33 | 7,35 | 7,27 | 7,33 | 0,08 |
| **T45** | - | 7,17 | 7,37 | 7,29 | 7,28 | 7,27 | 7,25 | 7,27 | 7,27 | 7,27 | 0,05 |
| **T75** | - | 7,20 | 7,26 | 7,28 | 7,26 | 7,29 | 7,19 | 7,25 | 7,24 | 7,24 | 0,04 |
| **T90** | - | 7,17 | 7,19 | 7,30 | 7,27 | 7,28 | 7,15 | 7,28 | 7,26 | 7,24 | 0,06 |
