# Supplementary material for: Anesthesia for non-obstetric surgery during late term pregnancy in mares
Source: PLoS One. 2024 Nov 22;19(11):e0313563. doi: 10.1371/journal.pone.0313563 (PMC11584139; doi:10.1371/journal.pone.0313563)
Supplement: S15 Table — Maternal Cardiac Output. Maternal cardiac output (L) during general inhalation anesthesia and dorsal recumbency of mares in the last month of gestation. (DOCX) [file pone.0313563.s015.docx]

**S15 Table. Raw Data. Maternal Cardiac Output.** Maternal cardiac output (L) during general inhalation anesthesia and dorsal recumbency of mares in the last month of gestation.

| **Cardiac Output (L)** | | | | | | | | | | | |
| --- | --- | --- | --- | --- | --- | --- | --- | --- | --- | --- | --- |
| **Time (minutes)** | **Horse 1** | **Horse 2** | **Horse 3** | **Horse 4** | **Horse 5** | **Horse 6** | **Horse 7** | **Horse 8** | **Horse 9** | **Mean** | **SD** |
| **T0** | - | 30,24 | 36,45 | 38,20 | 40,30 | 41,70 | 42,90 | 40,28 | 26,90 | 37,12 | 5,71 |
| **T15** | - | 47,28 | 21,60 | 30,00 | 30,60 | 44,20 | 16,50 | 19,62 | 19,20 | 28,63 | 11,73 |
| **T25** | - |  | 30,30 | 38,70 | 39,40 | 30,45 | 36,68 | 19,30 | 27,30 | 31,73 | 7,18 |
| **T35** | - | 45,60 | 36,40 | 38,40 | 46,20 | 32,10 | 53,33 | 23,40 | 28,58 | 38,00 | 10,00 |
| **T45** | - | 38,46 | 39,90 | 46,90 | 54,20 | 29,40 | 37,28 | 36,45 | 22,32 | 38,11 | 9,77 |
| **T60** | - | 39,00 | 27,00 | 38,60 | 41,90 | 33,42 | 43,10 | 29,85 | 15,60 | 33,56 | 9,22 |
| **T75** | - | 34,08 | 25,13 | 26,80 | 46,20 | 40,90 | 41,00 | 20,10 | 20,04 | 31,78 | 10,18 |
| **T90** | - | 42,30 | 22,90 | 26,30 | 34,30 | 25,40 | 35,82 | 24,40 | 20,90 | 29,04 | 7,52 |
