# Supplementary material for: Anesthesia for non-obstetric surgery during late term pregnancy in mares
Source: PLoS One. 2024 Nov 22;19(11):e0313563. doi: 10.1371/journal.pone.0313563 (PMC11584139; doi:10.1371/journal.pone.0313563)
Supplement: S16 Table — Maternal Pulmonary Artery Pressure. Maternal pulmonary artery pressure (mmHg) during general inhalation anesthesia and dorsal recumbency of mares in the last month of gestation. (DOCX) [file pone.0313563.s016.docx]

**S16 Table. Raw Data. Maternal Pulmonary Artery Pressure.** Maternal pulmonary artery pressure (mmHg) during general inhalation anesthesia and dorsal recumbency of mares in the last month of gestation.

| **Pulmonary Artery Pressure (mmHg)** | | | | | | | | | | | |
| --- | --- | --- | --- | --- | --- | --- | --- | --- | --- | --- | --- |
| **Time (minutes)** | **Horse 1** | **Horse 2** | **Horse 3** | **Horse 4** | **Horse 5** | **Horse 6** | **Horse 7** | **Horse 8** | **Horse 9** | **Mean** | **SD** |
| **T0** | - | 21 | - | 10 | 37 | 23 | 25 | 27 | 23 | 23,71 | 8,01 |
| **T15** | - | 9 | 12 | 9 | 8 | 18 | 8 | 7 | 16 | 10,88 | 4,09 |
| **T25** | - | 14 | 8 | 13 | 19 | 18 | 8 | 14 | 12 | 13,25 | 4,03 |
| **T35** | - | 10 | 7 | -6 | 22 | 1 | 5 | 14 | 12 | 8,13 | 8,51 |
| **T45** | - | 9 | 10 | 20 | 8 | 11 | 14 | 4 | 13 | 11,13 | 4,73 |
| **T60** | - | 8 | 12 | 9 | 2 | 4 | 16 | 4 | 14 | 8,63 | 5,10 |
| **T75** | - | 8 | 11 | 4 | 8 | 6 | 11 | 4 | 8 | 7,50 | 2,73 |
| **T90** | - | 10 | 15 | 2 | 1 | 5 | 7 | 15 | 7 | 7,75 | 5,31 |
