# Supplementary material for: Anesthesia for non-obstetric surgery during late term pregnancy in mares
Source: PLoS One. 2024 Nov 22;19(11):e0313563. doi: 10.1371/journal.pone.0313563 (PMC11584139; doi:10.1371/journal.pone.0313563)
Supplement: S17 Table — Maternal Central Venous Pressure. Maternal central venous pressure (mmHg) during general inhalation anesthesia and dorsal recumbency of mares in the last month of gestation. (DOCX) [file pone.0313563.s017.docx]

**S17 Table. Raw Data. Maternal Central Venous Pressure.** Maternal central venous pressure (mmHg) during general inhalation anesthesia and dorsal recumbency of mares in the last month of gestation.

| **Central Venous Pressure (mmHg)** | | | | | | | | | | | |
| --- | --- | --- | --- | --- | --- | --- | --- | --- | --- | --- | --- |
| **Time (minutes)** | **Horse 1** | **Horse 2** | **Horse 3** | **Horse 4** | **Horse 5** | **Horse 6** | **Horse 7** | **Horse 8** | **Horse 9** | **Mean** | **SD** |
| **T0** | - | 4 | - | 4 | 2 | 5 | 27 | 11 | 9 | 8,86 | 8,59 |
| **T15** | - | -8 | -7 | -3 | 1 | 8 | 19 | 1 | 2 | 1,63 | 8,72 |
| **T25** | - | - | -5 | -4 | 4 | -1 | 23 | 4 | -8 | 1,86 | 10,35 |
| **T35** | - | -6 | -2 | -6 | 18 | -2 | 22 | 1 | -10 | 1,88 | 11,72 |
| **T45** | - | -5 | -4 | -8 | 3 | -1 | 6 | 1 | -3 | -1,38 | 4,57 |
| **T60** | - | -7 | -3 | -7 | 2 | 0 | 1 | 5 | 1 | -1,00 | 4,31 |
| **T75** | - | -7 | -1 | -7 | 1 | -2 | 6 | -2 | 0 | -1,50 | 4,24 |
| **T90** | - | -6 | 0 | -3 | 3 | 4 | 17 | 0 | -2 | 1,63 | 6,99 |
