# Supplementary material for: Anesthesia for non-obstetric surgery during late term pregnancy in mares
Source: PLoS One. 2024 Nov 22;19(11):e0313563. doi: 10.1371/journal.pone.0313563 (PMC11584139; doi:10.1371/journal.pone.0313563)
Supplement: S18 Table — Maternal Stroke Volume. Maternal stroke volume (mL) during general inhalation anesthesia and dorsal recumbency of mares in the last month of gestation. (DOCX) [file pone.0313563.s018.docx]

**S18 Table. Raw Data. Maternal Stroke Volume.** Maternal stroke volume (mL) during general inhalation anesthesia and dorsal recumbency of mares in the last month of gestation.

| **Stroke Volume (mL)** | | | | | | | | | | | |
| --- | --- | --- | --- | --- | --- | --- | --- | --- | --- | --- | --- |
| **Time (minutes)** | **Horse 1** | **Horse 2** | **Horse 3** | **Horse 4** | **Horse 5** | **Horse 6** | **Horse 7** | **Horse 8** | **Horse 9** | **Mean** | **SD** |
| **T0** | - | - | - | - | - | - | - | - | - | - | - |
| **T15** | - | 788,00 | 583,78 | 652,17 | 536,84 | 866,67 | 571,24 | 306,09 | 480,00 | 598,10 | 175,26 |
| **T25** | - | 0,00 | 432,86 | 466,27 | 486,42 | 276,82 | 277,06 | 226,90 | 580,85 | 343,40 | 185,27 |
| **T35** | - | 592,21 | 438,55 | 426,67 | 531,03 | 373,26 | 393,62 | 208,22 | 571,50 | 441,88 | 124,91 |
| **T45** | - | 506,05 | 458,62 | 498,94 | 595,60 | 330,34 | 398,26 | 213,34 | 455,51 | 432,08 | 117,89 |
| **T75** | - | 405,71 | 405,24 | 326,83 | 563,41 | 459,55 | 360,13 | 192,94 | 477,14 | 398,87 | 110,89 |
| **T90** | - | 0,00 | 458,00 | 370,42 | 483,10 | 246,60 | 259,69 | 217,51 | 522,50 | 319,73 | 173,67 |
