# Supplementary material for: Anesthesia for non-obstetric surgery during late term pregnancy in mares
Source: PLoS One. 2024 Nov 22;19(11):e0313563. doi: 10.1371/journal.pone.0313563 (PMC11584139; doi:10.1371/journal.pone.0313563)
Supplement: S19 Table — Fetal heart rate. Fetal heart rate (bpm) at T0, during general inhalation anesthesia and dorsal recumbency of mares in the last month of gestation, and after maternal recovery from anesthesia. (DOCX) [file pone.0313563.s019.docx]

**S19 Table. Raw Data. Fetal heart rate.** Fetal heart rate (bpm) at T0, during general inhalation anesthesia and dorsal recumbency of mares in the last month of gestation, and after maternal recovery from anesthesia.

| **Fetal Heart Rate (bpm)** | | | | | | | | | | | |
| --- | --- | --- | --- | --- | --- | --- | --- | --- | --- | --- | --- |
| **Time (minutes)** | **Horse 1** | **Horse 2** | **Horse 3** | **Horse 4** | **Horse 5** | **Horse 6** | **Horse 7** | **Horse 8** | **Horse 9** | **Mean** | **SD** |
| **T0** | 90 | - | 99 | 86 | 70 | 100 | 70 | 87 | 74 | 84,50 | 12,07 |
| **T15** | - | 75 | 68 | 89 | 70 | 63 | 81 | 75 | 68 | 73,63 | 8,31 |
| **T25** | - | 68 | 63 | 75 | 62 | 55 | 63 | 75 | 60 | 65,13 | 7,08 |
| **T35** | - | 56 | 62 | 72 | 61 | 53 | 59 | 73 | 63 | 62,38 | 7,05 |
| **T45** | - | 60 | 59 | 72 | 61 | 52 | 58 | 67 | 74 | 62,88 | 7,49 |
| **T60** | - | 56 | 56 | 71 | 60 | 54 | 58 | 64 | 64 | 60,38 | 5,66 |
| **T75** | - | 56 | 58 | 74 | 59 | 56 | 58 | 63 | 62 | 60,75 | 5,92 |
| **T90** | 64 | - | - | - | - | - | - | - | - | 64,00 | - |
| **Tpost** | 88 | 67 | 66 | 80 | 89 | 70 | 77 | 107 | 59 | 78,11 | 14,82 |
