# Supplementary material for: Anesthesia for non-obstetric surgery during late term pregnancy in mares
Source: PLoS One. 2024 Nov 22;19(11):e0313563. doi: 10.1371/journal.pone.0313563 (PMC11584139; doi:10.1371/journal.pone.0313563)
Supplement: S20 Table — Maternal EtCO2. Maternal EtCO2 during general inhalation anesthesia and dorsal recumbency of mares in the last month of gestation. (DOCX) [file pone.0313563.s020.docx]

**S20 Table. Raw Data. Maternal EtCO_2_.** Maternal EtCO_2_ during general inhalation anesthesia and dorsal recumbency of mares in the last month of gestation.

| **EtCO_2_** | | | | | | | | | | | |
| --- | --- | --- | --- | --- | --- | --- | --- | --- | --- | --- | --- |
| **Time (minutes)** | **Horse 1** | **Horse 2** | **Horse 3** | **Horse 4** | **Horse 5** | **Horse 6** | **Horse 7** | **Horse 8** | **Horse 9** | **Mean** | **SD** |
| **T15** | 50 | 47 | 33 | 37 | 30 | 46 | 29 | 29 | 49 | 38,89 | 9,05 |
| **T25** | 45 | 49 | 35 | 44 | 32 | 35 | 39 | 53 | 37 | 41,00 | 7,12 |
| **T35** | 49 | 50 | 35 | 43 | 45 | 65 | 43 | 60 | 54 | 49,33 | 9,26 |
| **T45** | 49 | 38 | 41 | 46 | 52 | 54 | 43 | 59 | 41 | 47,00 | 7,00 |
| **T55** | 54 | 32 | 45 | 45 | 50 | 65 | 43 | 55 | 57 | 49,56 | 9,59 |
| **T65** | 52 | 32 | 47 | 46 | 50 | 50 | 53 | 58 | 47 | 48,33 | 7,16 |
| **T75** | 47 | 32 | 22 | 43 | 58 | 37 | 35 | 53 | 50 | 41,89 | 11,41 |
| **T85** | 55 | 31 | 28 | 45 | - | - | 53 | - | 48 | 43,33 | 11,33 |
| **T90** | - | - | 48 | 42 | 52 | 53 | - | 49 | 32 | 46,00 | 7,87 |
