# Supplementary material for: Anesthesia for non-obstetric surgery during late term pregnancy in mares
Source: PLoS One. 2024 Nov 22;19(11):e0313563. doi: 10.1371/journal.pone.0313563 (PMC11584139; doi:10.1371/journal.pone.0313563)
Supplement: S21 Table — Maternal SpO2. Maternal SpO2 during general inhalation anesthesia and dorsal recumbency of mares in the last month of gestation. (DOCX) [file pone.0313563.s021.docx]

**S21 Table. Raw Data. Maternal SpO_2_.** Maternal SpO_2_ during general inhalation anesthesia and dorsal recumbency of mares in the last month of gestation.

| **SpO_2_** | | | | | | | | | | | |
| --- | --- | --- | --- | --- | --- | --- | --- | --- | --- | --- | --- |
| **Time (minutes)** | **Horse 1** | **Horse 2** | **Horse 3** | **Horse 4** | **Horse 5** | **Horse 6** | **Horse 7** | **Horse 8** | **Horse 9** | **Mean** | **SD** |
| **T15** | 91 | 90 | 95 | 96 | 98 | 96 | 98 | 95 | 97 | 95,11 | 2,85 |
| **T25** | 91 | 93 | 99 | 98 | 98 | 100 | 94 | 97 | 98 | 96,44 | 3,05 |
| **T35** | 93 | 95 | 99 | 99 | 98 | 99 | 93 | 96 | 98 | 96,67 | 2,50 |
| **T45** | 91 | 95 | 100 | 99 | 98 | 100 | 98 | 98 | 99 | 97,56 | 2,88 |
| **T55** | 92 | 97 | 100 | 98 | 98 | 99 | 98 | 98 | 99 | 97,67 | 2,29 |
| **T65** | 93 | 99 | 99 | 98 | 98 | 100 | 98 | 98 | 98 | 97,89 | 1,96 |
| **T75** | 91 | 99 | 100 | 99 | 98 | 99 | 98 | 98 | 99 | 97,89 | 2,67 |
| **T85** | 90 | 97 | 99 | 98 | - | - | 97 | - | 98 | 96,50 | 3,27 |
| **T90** | - | - | 99 | 98 | 98 | 99 | - | 97 | 98 | 98,17 | 0,75 |
