# Supplementary material for: Anesthesia for non-obstetric surgery during late term pregnancy in mares
Source: PLoS One. 2024 Nov 22;19(11):e0313563. doi: 10.1371/journal.pone.0313563 (PMC11584139; doi:10.1371/journal.pone.0313563)
Supplement: S22 Table — Maternal Base Excess. Maternal base excess (mmol/L) during general inhalation anesthesia and dorsal recumbency of mares in the last month of gestation. (DOCX) [file pone.0313563.s022.docx]

**S22 Table. Raw Data. Maternal Base Excess.** Maternal base excess (mmol/L) during general inhalation anesthesia and dorsal recumbency of mares in the last month of gestation.

| **Base Excess (mmol/L)** | | | | | | | | | | | |
| --- | --- | --- | --- | --- | --- | --- | --- | --- | --- | --- | --- |
| **Time (minutes)** | **Horse 1** | **Horse 2** | **Horse 3** | **Horse 4** | **Horse 5** | **Horse 6** | **Horse 7** | **Horse 8** | **Horse 9** | **Mean** | **SD** |
| **T15** | - | -6 | -1 | 3 | 2 | 4 | -3 | 4 | 1 | 0,50 | 3,59 |
| **T45** | - | -8 | 1 | 4 | 2 | 3 | -3 | 1 | 1 | 0,13 | 3,87 |
| **T75** | - | -8 | -1 | 2 | 3 | 3 | -3 | 2 | 1 | -0,13 | 3,80 |
| **T90** | - | -10 | -2 | 3 | 0 | 4 | -6 | -2 | -1 | -1,75 | 4,56 |
