# Supplementary material for: Anesthesia for non-obstetric surgery during late term pregnancy in mares
Source: PLoS One. 2024 Nov 22;19(11):e0313563. doi: 10.1371/journal.pone.0313563 (PMC11584139; doi:10.1371/journal.pone.0313563)
Supplement: S23 Table — Maternal HCO3. Maternal HCO3 during general inhalation anesthesia and dorsal recumbency of mares in the last month of gestation. (DOCX) [file pone.0313563.s023.docx]

**S23 Table. Raw Data. Maternal HCO_3_.** Maternal HCO_3_ during general inhalation anesthesia and dorsal recumbency of mares in the last month of gestation.

| **HCO_3_ (mmol/L)** | | | | | | | | | | | |
| --- | --- | --- | --- | --- | --- | --- | --- | --- | --- | --- | --- |
| **Time (minutes)** | **Horse 1** | **Horse 2** | **Horse 3** | **Horse 4** | **Horse 5** | **Horse 6** | **Horse 7** | **Horse 8** | **Horse 9** | **Mean** | **SD** |
| **T15** | - | 22,7 | 24,3 | 28,5 | 25,3 | 29,8 | 23 | 29,2 | 27,8 | 26,33 | 2,84 |
| **T45** | - | 20,7 | 26,5 | 30,5 | 28,3 | 29,6 | 24,2 | 28,2 | 28,1 | 27,01 | 3,19 |
| **T75** | - | 20,5 | 25,9 | 29 | 29,7 | 30,2 | 24,8 | 29,3 | 29,2 | 27,33 | 3,36 |
| **T90** | - | 18,8 | 26,7 | 29,6 | 26,9 | 30,8 | 22,8 | 24,8 | 26,7 | 25,89 | 3,80 |
