# Supplementary material for: Anesthesia for non-obstetric surgery during late term pregnancy in mares
Source: PLoS One. 2024 Nov 22;19(11):e0313563. doi: 10.1371/journal.pone.0313563 (PMC11584139; doi:10.1371/journal.pone.0313563)
Supplement: S24 Table — Maternal tCO2. Maternal tCO2 during general inhalation anesthesia and dorsal recumbency of mares in the last month of gestation. (DOCX) [file pone.0313563.s024.docx]

**S24 Table. Raw Data. Maternal tCO_2_.** Maternal tCO_2_ during general inhalation anesthesia and dorsal recumbency of mares in the last month of gestation.

| **tCO_2_ (mmol/L)** | | | | | | | | | | | |
| --- | --- | --- | --- | --- | --- | --- | --- | --- | --- | --- | --- |
| **Time (minutes)** | **Horse 1** | **Horse 2** | **Horse 3** | **Horse 4** | **Horse 5** | **Horse 6** | **Horse 7** | **Horse 8** | **Horse 9** | **Mean** | **SD** |
| **T15** | - | 25 | 26 | 30 | 26 | 31 | 24 | 31 | 30 | 27,88 | 2,90 |
| **T45** | - | 22 | 28 | 32 | 30 | 32 | 26 | 30 | 30 | 28,75 | 3,37 |
| **T75** | - | 22 | 28 | 31 | 32 | 32 | 27 | 31 | 31 | 29,25 | 3,45 |
| **T90** | - | 20 | 29 | 31 | 29 | 33 | 25 | 26 | 29 | 27,75 | 4,03 |
