# Supplementary material for: Anesthesia for non-obstetric surgery during late term pregnancy in mares
Source: PLoS One. 2024 Nov 22;19(11):e0313563. doi: 10.1371/journal.pone.0313563 (PMC11584139; doi:10.1371/journal.pone.0313563)
Supplement: S25 Table — Maternal SO2. Maternal SO2 during general inhalation anesthesia and dorsal recumbency of mares in the last month of gestation. (DOCX) [file pone.0313563.s025.docx]

**S25 Table. Raw Data. Maternal SO_2_.** Maternal SO_2_ during general inhalation anesthesia and dorsal recumbency of mares in the last month of gestation.

| **SO_2_ (%)** | | | | | | | | | | | |
| --- | --- | --- | --- | --- | --- | --- | --- | --- | --- | --- | --- |
| **Time (minutes)** | **Horse 1** | **Horse 2** | **Horse 3** | **Horse 4** | **Horse 5** | **Horse 6** | **Horse 7** | **Horse 8** | **Horse 9** | **Mean** | **SD** |
| **T15** | - | 76 | 99 | 88 | 98 | 99 | 92 | 86 | 90 | 91,00 | 7,91 |
| **T45** | - | 80 | 100 | 97 | 99 | 99 | 98 | 95 | 96 | 95,50 | 6,48 |
| **T75** | - | 98 | 100 | 99 | 99 | 100 | 91 | 94 | 93 | 96,75 | 3,54 |
| **T90** | - | 97 | 99 | 99 | 99 | 100 | 88 | 93 | 91 | 95,75 | 4,50 |
