# Supplementary material for: Anesthesia for non-obstetric surgery during late term pregnancy in mares
Source: PLoS One. 2024 Nov 22;19(11):e0313563. doi: 10.1371/journal.pone.0313563 (PMC11584139; doi:10.1371/journal.pone.0313563)
Supplement: S26 Table — Maternal Sodium. Maternal sodium (mmol/L) during general inhalation anesthesia and dorsal recumbency of mares in the last month of gestation. (DOCX) [file pone.0313563.s026.docx]

**S26 Table. Raw Data. Maternal Sodium.** Maternal sodium (mmol/L) during general inhalation anesthesia and dorsal recumbency of mares in the last month of gestation.

| **Sodium (mmol/L)** | | | | | | | | | | | |
| --- | --- | --- | --- | --- | --- | --- | --- | --- | --- | --- | --- |
| **Time (minutes)** | **Horse 1** | **Horse 2** | **Horse 3** | **Horse 4** | **Horse 5** | **Horse 6** | **Horse 7** | **Horse 8** | **Horse 9** | **Mean** | **SD** |
| **T15** | - | 139 | 141 | 140 | 138 | 138 | 140 | 137 | 137 | 138,75 | 1,49 |
| **T45** | - | 136 | 139 | 139 | 137 | 136 | 138 | 138 | 134 | 137,13 | 1,73 |
| **T75** | - | 136 | 140 | 137 | 137 | 134 | 137 | 137 | 133 | 136,38 | 2,13 |
| **T90** | - | 134 | 139 | 138 | 137 | 134 | 136 | 136 | 133 | 135,88 | 2,10 |
