# Supplementary material for: Anesthesia for non-obstetric surgery during late term pregnancy in mares
Source: PLoS One. 2024 Nov 22;19(11):e0313563. doi: 10.1371/journal.pone.0313563 (PMC11584139; doi:10.1371/journal.pone.0313563)
Supplement: S27 Table — Maternal Potassium. Maternal potassium (mmol/L) during general inhalation anesthesia and dorsal recumbency of mares in the last month of gestation. (DOCX) [file pone.0313563.s027.docx]

**S27 Table. Raw Data. Maternal Potassium.** Maternal potassium (mmol/L) during general inhalation anesthesia and dorsal recumbency of mares in the last month of gestation.

| **Potassium (mmol/L)** | | | | | | | | | | | |
| --- | --- | --- | --- | --- | --- | --- | --- | --- | --- | --- | --- |
| **Time (minutes)** | **Horse 1** | **Horse 2** | **Horse 3** | **Horse 4** | **Horse 5** | **Horse 6** | **Horse 7** | **Horse 8** | **Horse 9** | **Mean** | **SD** |
| **T15** | - | 3,6 | 3 | 3,5 | 3,5 | 3,6 | 3,4 | 3,7 | 3,6 | 3,49 | 0,22 |
| **T45** | - | 3,2 | 3 | 3,1 | 3,2 | 3,5 | 3,1 | 3 | 3,6 | 3,21 | 0,22 |
| **T75** | - | 3,1 | 2,9 | 3,1 | 3,4 | 3,5 | 3,4 | 3,3 | 3,6 | 3,29 | 0,24 |
| **T90** | - | 3,1 | 2,9 | 3,2 | 3,4 | 3,4 | 3,2 | 3,1 | 3,6 | 3,24 | 0,22 |
