# Supplementary material for: Anesthesia for non-obstetric surgery during late term pregnancy in mares
Source: PLoS One. 2024 Nov 22;19(11):e0313563. doi: 10.1371/journal.pone.0313563 (PMC11584139; doi:10.1371/journal.pone.0313563)
Supplement: S28 Table — Maternal Calcium. Maternal calcium (mmol/L) during general inhalation anesthesia and dorsal recumbency of mares in the last month of gestation. (DOCX) [file pone.0313563.s028.docx]

**S28 Table. Raw Data. Maternal Calcium.** Maternal calcium (mmol/L) during general inhalation anesthesia and dorsal recumbency of mares in the last month of gestation.

| **Calcium (mmol/L)** | | | | | | | | | | | |
| --- | --- | --- | --- | --- | --- | --- | --- | --- | --- | --- | --- |
| **Time (minutes)** | **Horse 1** | **Horse 2** | **Horse 3** | **Horse 4** | **Horse 5** | **Horse 6** | **Horse 7** | **Horse 8** | **Horse 9** | **Mean** | **SD** |
| **T15** | - | 1,09 | 0,84 | 1,2 | 0,84 | 0,93 | 0,86 | 0,89 | 0,94 | 0,95 | 0,13 |
| **T45** | - | 0,91 | 0,85 | 1,07 | 0,84 | 0,9 | 0,84 | 0,48 | 0,79 | 0,84 | 0,17 |
| **T75** | - | 0,8 | 0,71 | 1,03 | 0,97 | 0,89 | 1,05 | 0,72 | 0,82 | 0,87 | 0,13 |
| **T90** | - | 0,82 | 0,83 | 0,97 | 0,82 | 1,04 | 0,77 | 0,51 | 0,77 | 0,82 | 0,16 |
