# Supplementary material for: Anesthesia for non-obstetric surgery during late term pregnancy in mares
Source: PLoS One. 2024 Nov 22;19(11):e0313563. doi: 10.1371/journal.pone.0313563 (PMC11584139; doi:10.1371/journal.pone.0313563)
Supplement: S29 Table — Maternal Lactate. Maternal lactate (mmol/L) during general inhalation anesthesia and dorsal recumbency of mares in the last month of gestation. (DOCX) [file pone.0313563.s029.docx]

**S29 Table. Raw Data. Maternal Lactate.** Maternal lactate (mmol/L) during general inhalation anesthesia and dorsal recumbency of mares in the last month of gestation.

| **Lactate (mmol/L)** | | | | | | | | | | | |
| --- | --- | --- | --- | --- | --- | --- | --- | --- | --- | --- | --- |
| **Time (minutes)** | **Horse 1** | **Horse 2** | **Horse 3** | **Horse 4** | **Horse 5** | **Horse 6** | **Horse 7** | **Horse 8** | **Horse 9** | **Mean** | **SD** |
| **T15** | - | 2,1 | 5,7 | 3,8 | 3,1 | 2,1 | 0,9 | 3,8 | 3,6 | 3,14 | 1,45 |
| **T45** | - | 2,1 | 4,9 | 2,7 | 1,7 | 2,4 | 4,6 | 3,8 | 4,4 | 3,33 | 1,25 |
| **T75** | - | 3,4 | 4,3 | 2,9 | 2,4 | 3 | 4 | 2 | 5 | 3,38 | 1,01 |
| **T90** | - | 3,8 | 3,9 | 3,1 | 3,4 | 3,2 | 4,9 | 2,3 | 2,8 | 3,43 | 0,79 |
