# Supplementary material for: Anesthesia for non-obstetric surgery during late term pregnancy in mares
Source: PLoS One. 2024 Nov 22;19(11):e0313563. doi: 10.1371/journal.pone.0313563 (PMC11584139; doi:10.1371/journal.pone.0313563)
Supplement: S30 Table — Maternal Cardiac Index. Maternal cardiac index (mL) during general inhalation anesthesia and dorsal recumbency of mares in the last month of gestation. (DOCX) [file pone.0313563.s030.docx]

**S30 Table. Raw Data. Maternal Cardiac Index.** Maternal cardiac index (mL) during general inhalation anesthesia and dorsal recumbency of mares in the last month of gestation.

| **Cardiac Index (mL)** | | | | | | | | | | | |
| --- | --- | --- | --- | --- | --- | --- | --- | --- | --- | --- | --- |
| **Time (minutes)** | **Horse 1** | **Horse 2** | **Horse 3** | **Horse 4** | **Horse 5** | **Horse 6** | **Horse 7** | **Horse 8** | **Horse 9** | **Mean** | **SD** |
| **T0** | - | 86,40 | 91,13 | 95,50 | 95,95 | 112,70 | 102,14 | 95,89 | 65,61 | 93,17 | 13,59 |
| **T15** | - | 135,09 | 54,00 | 75,00 | 72,86 | 119,46 | 39,29 | 46,71 | 46,83 | 73,65 | 35,63 |
| **T25** | - |  | 75,75 | 96,75 | 93,81 | 82,30 | 87,32 | 45,95 | 66,59 | 78,35 | 17,65 |
| **T35** | - | 130,29 | 91,00 | 96,00 | 110,00 | 86,76 | 126,96 | 55,71 | 69,70 | 95,80 | 26,06 |
| **T45** | - | 109,89 | 99,75 | 117,25 | 129,05 | 79,46 | 88,75 | 86,79 | 54,44 | 95,67 | 23,57 |
| **T60** | - | 111,43 | 67,50 | 96,50 | 99,76 | 90,32 | 102,62 | 71,07 | 38,05 | 84,66 | 24,18 |
| **T75** | - | 97,37 | 62,81 | 67,00 | 110,00 | 110,54 | 97,62 | 47,86 | 48,88 | 80,26 | 26,49 |
| **T90** | - | 120,86 | 57,25 | 65,75 | 81,67 | 68,65 | 85,29 | 58,10 | 50,98 | 73,57 | 22,49 |
