# Supplementary material for: Anesthesia for non-obstetric surgery during late term pregnancy in mares
Source: PLoS One. 2024 Nov 22;19(11):e0313563. doi: 10.1371/journal.pone.0313563 (PMC11584139; doi:10.1371/journal.pone.0313563)
Supplement: S31 Table — Maternal Peripheric Vascular Resistance. Maternal peripheric vascular resistance during general inhalation anesthesia and dorsal recumbency of mares in the last month of gestation. (DOCX) [file pone.0313563.s031.docx]

**S31 Table. Raw Data. Maternal Peripheric Vascular Resistance.** Maternal peripheric vascular resistance during general inhalation anesthesia and dorsal recumbency of mares in the last month of gestation.

| **Peripheric Vascular Resistance** | | | | | | | | | | | |
| --- | --- | --- | --- | --- | --- | --- | --- | --- | --- | --- | --- |
| **Time (minutes)** | **Horse 1** | **Horse 2** | **Horse 3** | **Horse 4** | **Horse 5** | **Horse 6** | **Horse 7** | **Horse 8** | **Horse 9** | **Mean** | **SD** |
| **T0** | - | - | - | - | - | - | - | - | - | - | - |
| **T15** | - | 84,50 | 196,05 | 122,51 | 101,83 | 65,08 | - | 171,04 | 183,10 | 132,02 | 51,60 |
| **T25** | - | - | 171,40 | 132,13 | 121,68 | 133,82 | 23,96 | 194,58 | 175,60 | 136,17 | 56,32 |
| **T35** | - | 110,39 | 136,09 | 135,25 | 65,72 | 144,37 | 40,46 | 180,97 | 195,73 | 126,12 | 52,92 |
| **T45** | - | 114,26 | 118,15 | 103,92 | 86,98 | 152,19 | 109,32 | 131,52 | 204,05 | 127,55 | 36,40 |
| **T75** | - | 161,77 | 181,27 | 190,81 | 112,41 | 111,35 | 99,39 | 234,53 | 231,25 | 165,35 | 53,60 |
| **T90** | - | - | 223,30 | 188,36 | 132,78 | 147,85 | - | 189,93 | 210,26 | 182,08 | 35,21 |
|  |  |  |  |  |  |  |  |  |  |  |  |
