# Supplementary material for: Anesthesia for non-obstetric surgery during late term pregnancy in mares
Source: PLoS One. 2024 Nov 22;19(11):e0313563. doi: 10.1371/journal.pone.0313563 (PMC11584139; doi:10.1371/journal.pone.0313563)
Supplement: Attachment — Submitted filename: Revisao PLOS ONE.docx [file pone.0313563.s032.docx]

Dear Editor Negrini Medeiros.

Follows bellows my review comments for the authors

1) Page 3 line 48 - Correct de reference list.

The authors make multiple statements in this paragraph, citing 3 references at the end of the paragraph. I believe it is more appropriate to cite each reference after each specific statement. If they don’t have any reference supporting each statement, it should be removed from the text

2) Page 3 line 56 - Asa 6 does not define anesthetic risk

Classifying a patient as ASA 6 doesn’t not imply any risk. ASA 6 patients are the ones with brain death, usually organ donors. The ASA classification stratified anesthetic risk from 1 to 5. Please, review this statement.

3) I believe all this part of the paragraph should be removed

(from page 3 line 54 to page 3 line 59). I don’t understand why the authors initiate this paragraph mentioning the importance of oxygenation to maternal and fetus well being, and after that shift to the ASA classification.

I also believe the lines 60 to 68 on page 3 should be moved to the previous paragraph, since it is the logical consequence of the discussion regarding oxygenation and maternal - fetus well being. Consequently, the third paragraph should start with: “The objective of this study…."

5) page 4 line 64 - There is no comparison, only description.

I don't believe the primary aim of the authors was comparison, since they don’t use controls. The main aim do the study should be rephrased to “describe”, instead of “compare".

7) Page 15 lince 252 - Show the correlation figures.

I really don’t understand why the authors does not show probably the most relevant figure at the results section of the manuscript. Surely all the plots describing the data are relevant. However, the person correlation between “maternal heart rate”, “arterial PH” and “fetus heart rate”are definitely the most relevant information, especially because of the very meaningful statistical association.

8) Page 16 line 261 - Is it really the first? This statement looks a little bit presumptuous

I really believe it super risk to make assumptions like that in a manuscript. Why not rephrase this statement to: “As far as we know, our paper was one of the first to…"

9) page 16 line 262 - The results does not support this conclusion.

I don't believe the results support concluding a progressive worsening of hemodynamic variables continuously during general anesthesia. We observe a initial worsening, which stabilizes after the first minutes after induction.

10) Page 17 line 285 - The readers deserve a little bit of explanation here.

I believe the authors should make it more clear to readers why it iso difficult to manage PaCO2 during anesthesia in a recumbent position.

11) page 18 line 329 - Unnecessary explanation here.

I believe this theoretical explanation regarding pre load does not belong in the article. It might belong to a book, defiantly not in a article. I suggest removing this part.

12) Page 21 libre 390 - State the limitation imposed by the lack of controls.

The authors should highlight as maybe the most important limitation of their study the lack of comparison with a control group.

13) Page 22 line 393 - I believe the conclusion has a lot of room for improvement. The authors should try something like that..

"The present study indicates the general inhalation anesthesia in late term pregnancy in mares in a recumbent position implies in significant hemodynamic and metabolic changes. Up to 90 minutes those changes does not seem to affect negatively the maternal-fetus prognosis. However, further studies should be conducted, with bigger sample sizes and comparison with controls, to support this conclusion.”

I appreciate the opportunity of reviewing this manuscript for PLOS ONE.

Best

Tatiana P.C. Linhares

Rio de Janeiro, 07/23/2024
