# Supplementary material for: Anesthesia for non-obstetric surgery during late term pregnancy in mares
Source: PLoS One. 2024 Nov 22;19(11):e0313563. doi: 10.1371/journal.pone.0313563 (PMC11584139; doi:10.1371/journal.pone.0313563)
Supplement: Attachment — Submitted filename: Response to reviewers.docx [file pone.0313563.s033.docx]

Dear editor and reviewers,

Thank you very much for the attention and the chance of publishing an article in so important journal. We took care of all the comments and suggestions that the reviewers had done carefully and we made all the changes suggested and respond all the comments individually. All the changes we have done in the manuscript are highlighted (yellow).

So, we hope the preparation of this revision will permit a positive final decision on my report's suitability for publication in Plos One.

Thank you very much for the additional comments.

Best regards.

Renata G S Dória et al.

1. We have ensured that our manuscript meets PLOS ONE's style requirements, as suggested.

2. We need to include this paragraph, as suggested. Please include this amended for us.

This research was funded by São Paulo Research Foundation (FAPESP) grants number [2020/09633-0 CAAV, 2020/01387-0 PHSB, and 2021/13378-8 RGSD], and Technological Development (CNPq) grant number [309701/2022-8 RGSD]. And the APC was funded by [FAPESP; grants number 2020/09633-0 CAAV, 2020/01387-0 PHSB, and 2021/13378-8 RGSD]. This study was financed in part by the Coordenação de Aperfeiçoamento de Pessoal de Nível Superior – Brasil (CAPES) – Finance Code 001. The funders had no role in study design, data collection and analysis, decision to publish, or preparation of the manuscript.

3. We have included captions for our Supporting Information files at the end of our manuscript, and update the in-text citations to match accordingly.

Dear Editor,

We have taken careful consideration on the comments made by both reviewers. Additionally, we were more cautious to conclude what can be supported by our results, as suggested.

We removed the term “comparison” in the text, as suggested. And we added the lack of a control group as an important limitation of our work.

Reviewer #1: I believe all my relevant comments are already in the attached document. The data presented by the authors are interesting. However, I believe they haven't highlighted the most relevant data. Moreover, the discussion should be revised in a way that it reflects what is shown at the results section. Additionally, the results also does not support the authors conclusions, and limitations should also be revised.

**Answer: Suggestions accepted. We have corrected the manuscript, as suggested in the attached document and highlighted the change in the text (yellow).**

Reviewer #2: Statistic analysis should be clearly described in the revised manuscript. The authors stated that they had performed comparisons. However, they only reported linear correlation coefficients. Conclusions did not derive from the statistical significance of the data. Moreover, correction for multiple comparisons were not described.

**Answer: The statistical analysis was clearly described by a statistic professor (Ghantous, G.F.). We did not make comparisons. This word was removed from the text. We inserted the correlations table (Pearson correlation) inside the text to make it easier to understand. We corrected the conclusion statement.**

1) Page 3 line 48 - Correct de reference list.

**Answer: Corrected, as suggested.**

2) Page 3 line 56 - Asa 6 does not define anesthetic risk

**Answer: Corrected, as suggested.**

3) I believe all this part of the paragraph should be removed

**Answer: The paragraph was removed and the next paragraph was moved to the previous, as suggested.**

4) page 4 line 64 - There is no comparison, only description.

**Answer: Corrected, as suggested.**

5) Page 15 lince 252 - Show the correlation figures.

**Answer: Corrected, as suggested. The supplementary file was inserted in the text.**

6) Page 16 line 261 - Is it really the first? This statement looks a little bit presumptuous

**Answer: Corrected, as suggested.**

7) page 16 line 262 - The results does not support this conclusion.

**Answer: Corrected, as suggested. The paragraph was removed.**

10) Page 17 line 285 - The readers deserve a little bit of explanation here.

**Answer: Corrected, as suggested. We added the suggested explanation in the manuscript** (why it iso difficult to manage PaCO2 during anesthesia in a recumbent position).

11) page 18 line 329 - Unnecessary explanation here.

**Answer: Corrected, as suggested. The paragraph was removed.**

12) Page 21 libre 390 - State the limitation imposed by the lack of controls.

**Answer: Corrected, as suggested. This limitation was** highlighted**.**

13) Page 22 line 393 - I believe the conclusion has a lot of room for improvement. The authors should try something like that

**Answer: Corrected, as suggested.**
